# Hematuria following arginine growth hormone stimulation testing in a child: a case report and literature review

**DOI:** 10.3389/fped.2026.1763581

**Published:** 2026-04-09

**Authors:** Jiayang Song, Rong Zhao, Pan Li, Yong Liu, Zhongfu Tan

**Affiliations:** 1 Department of Pharmacy, Cangxi People's Hospital, Guangyuan, Sichuan, China; 2 Department of Pediatrics, Cangxi People's Hospital, Guangyuan, Sichuan, China; 3 Department of Medical Affairs, Cangxi People's Hospital, Guangyuan, Sichuan, China

**Keywords:** arginine, case report, growth hormone stimulation testing, hematuria, review of the literature

## Abstract

**Background:**

Arginine growth hormone (GH) testing is a commonly used and generally safe pediatric procedure for assessing GH deficiency. The occurrence of gross hematuria following this testing is a rare adverse drug reaction that had not been previously encountered at our institution, raising significant concerns among both patients and clinicians.

**Case presentation:**

An 11-year-and-3-month-old boy presented with short stature. Approximately 7 h after undergoing a combined arginine and levodopa GH stimulation testing, he developed painful gross hematuria accompanied by blood clots. A subsequent comprehensive clinical evaluation ruled out other potential etiologies, leading to the diagnosis of drug-induced hematuria. The patient was treated with urine alkalinization, and the gross hematuria substantially resolved within 8 days. Furthermore, a review of the literature identified 9 case reports involving 15 patients who developed hematuria following arginine GH stimulation testing. The majority of these patients were male, with a broad age distribution. Most individuals presented solely with a change in urine color; hematuria typically manifested within 1–3 days post-administration and resolved spontaneously within approximately 1 week. Standard management strategies primarily include urine alkalinization and fluid supplementation. Current studies hypothesize that the underlying mechanism may involve drug-induced alterations in the permeability of the glomerular filtration membrane or a triggered immune response.

**Conclusions:**

Hematuria induced by GH stimulation testing is a rare but noteworthy adverse drug reaction. Therefore, prior to initiating the test, clinicians must thoroughly inform patients and their families of this potential risk to alleviate unnecessary anxiety, and closely monitor for the possible onset of hematuria post-testing.

## 1 Introduction

Growth hormone (GH) deficiency (GHD) is an endocrine and metabolic disorder caused by insufficient secretion of GH from the anterior pituitary gland, with short stature being its primary clinical manifestation (1). GH stimulation testing is routinely used to assess GHD. Common pharmacological agents employed in these tests include arginine, clonidine, and levodopa (2). Arginine stimulates GH secretion by inhibiting somatostatin tone and activating alpha-adrenergic receptors (3). Although nausea and vomiting are common adverse reactions to arginine, the medications used in GH stimulation tests generally have a favorable safety profile (4).

In this report, we present a case of hematuria associated with the combined administration of arginine and levodopa. Notably, this adverse reaction is not listed in the drug package inserts for either arginine or levodopa in China. This represents a highly rare adverse drug reaction, which caused significant concern among the clinical team and the patient's family.

## 2 Case presentation

An 11-year-and-3-month-old boy presented to our facility for the evaluation of short stature. He had a history of noticeable growth retardation for over 2 years, with a height significantly lower than that of his age-matched peers; however, his exact historical growth velocity was undocumented. Initial evaluation revealed no pubic or axillary hair and no signs of gynecomastia. He had no digital anomalies, and his visual and hearing functions were intact. His dietary habits were normal, with no selective eating behaviors reported. His sleep duration and quality were adequate, his physical activity level was moderate, and his academic performance was satisfactory. He was subsequently admitted to the hospital with a primary diagnosis of short stature.

Upon admission, physical examination revealed the following: height, 132.5 cm; sitting height, 73.5 cm; weight, 29 kg; and body mass index (BMI), 16.5 kg/m^2^. His vital signs were stable, with a blood pressure of 105/68 mmHg and a respiratory rate of 18 breaths/min. The patient was conscious and alert, exhibiting a normal nutritional status and well-proportioned limbs. No dysmorphic facial features were noted. Body posture, palmar creases, and hair distribution across the head, chest, abdomen, and extremities were all unremarkable. No café-au-lait macules were observed. Genital examination indicated early pubertal development of the scrotum and penis. Examinations of the remaining systems revealed no other abnormalities.

The patient had an unremarkable past medical and family history. Upon admission, a complete blood count and urinalysis were normal. Liver and renal function tests, thyroid function, hepatitis B serology, insulin, vitamin D, and baseline GH levels were all within normal limits. Doppler ultrasonography of the bilateral adrenal glands and magnetic resonance imaging (MRI) of the pituitary gland revealed no structural abnormalities.

A GH stimulation testing was performed at approximately 11:00 a.m. on the day of admission. The pharmacological protocol consisted of an immediate oral dose of levodopa (0.29 g), combined with an intravenous infusion of arginine hydrochloride (14.5 g diluted in 87 mL of sterile water for injection) administered at a rate of 50 drops/min. Prior to drug administration, the patient was non-fasting and allowed *ad libitum* water intake. Serum GH levels measured at 30, 60, 90, and 120 min post-administration were 6.77, 3.10, 1.88, and 0.55 ng/mL, respectively.

On the evening of the stimulation test, the patient noted a change in urine color, presenting as gross total hematuria accompanied by dysuria. As the symptoms persisted for a day without improvement, he returned to our outpatient department the following evening. Urinalysis and urine sediment analysis revealed positive results for ketones (+), occult blood (+), protein (+), and white blood cells (+), alongside a markedly elevated red blood cell (RBC) count of 10,820/μL (reference range: 0–17/μL). Macroscopic blood clots were also visible in the urine. Doppler ultrasonography of the kidneys, bladder, and prostate showed no remarkable structural abnormalities. Consequently, a diagnosis of gross hematuria was established. Empirical treatment with oral carbazochrome (2.5 mg three times daily for 14 days, The actual medication usage is unclear) was initiated to reduce capillary permeability and mitigate urinary tract bleeding. On day 3, a urine bacterial culture returned negative. The patient subsequently sought a second opinion at another institution, where comprehensive evaluations failed to identify any underlying urological diseases or infectious etiologies. Although gross hematuria initially persisted, urinary alkalinization therapy was administered during this period. After 8 days, the urine color returned to normal, and the patient was discharged. Over three consecutive months of telephone follow-up, the patient reported no recurrence of macroscopic hematuria. The longitudinal changes in relevant urinary laboratory parameters before and after the hematuria episode are summarized in Table 1.

**Table 1:** Changes in urine-related laboratory parameters before and after hematuria.

Time	Events	Urine-related indicator results
On Day 1	Baseline (pre-arginine + levodopa)	Urinalysis/urine sediment test indicates: occult blood: (−); protein: (−); color: light yellow; clarity: clear; [urobilinogen: normal 3.4 µmol/L; bilirubin: (−); ketones: (−); nitrite: (−); leukocytes: (−); glucose: (−); urine specific gravity: 1.015 (all subsequent indicators are normal)]
On Day 1	Red urine appeared on the night of taking the medication, accompanied by painful urination	Observation
On the evening of Day 2	Red urine and dysuria, lasting for 1 day.	(1) Outpatient urinalysis and urine sediment analysis showed: ketones (+), occult blood (+), protein (+), white blood cells (+), and red blood cells 10,820/μL (reference range: 0–17). Blood clots were visibly present in the urine. (2) Color Doppler ultrasound revealed no definite abnormalities in the kidneys, bladder, or prostate.
On Day 3	Persistent red urine	urine bacterial culture results: (−)
On Day 4	Persistent red urine	Mean corpuscular volume (MCV):84.2 fL↑
On Day 8	The red urine has basically disappeared	Outpatient urinalysis and urine sediment analysis showed: red blood cells 10,820/μL↑, occult blood (+++), protein (++), color: light yellow, clarity: slightly cloudy
On Day 10	The urine is light yellow, and the red urine has disappeared.	Outpatient urinalysis and urine sediment analysis showed: red blood cells 174/μL↑, occult blood (++), protein (+), color: light yellow, clarity: clear
On Day 11	Pale yellow urine	Outpatient urinalysis and urine sediment analysis showed: red blood cells 156/μL↑, occult blood (++), protein (−), color: light yellow, clarity: clear
On Day 12 (the first time)	Pale yellow urine	Outpatient urinalysis and urine sediment analysis showed: red blood cells 15/μL↑, occult blood (+), protein (−), color: light yellow, clarity: clear
On Day 12 (4 h after the first time)	Pale yellow urine	Outpatient urinalysis and urine sediment analysis showed: red blood cells 0/μL↑, occult blood (−), protein (−), color: light yellow, clarity: clear

## 3 Discussion

### 3.1 Diagnosis of exclusion

Based on the clinical history, the patient reported normal urine color prior to the onset of hematuria, with no preceding symptoms of dysuria, urinary frequency, or urgency. He reported no recent upper respiratory tract infections or febrile illnesses, had an unremarkable past medical history, and had not taken any medications other than the administered levodopa and arginine hydrochloride. Furthermore, there was no recent history of strenuous exercise or trauma.

On the day of admission, prior to the GH stimulation testing involving arginine and levodopa, the patient's baseline assessments were unremarkable. Routine blood tests, urinalysis, as well as hepatic and renal function panels, were all normal. Thyroid function, hepatitis B serology, insulin, vitamin D, and baseline GH levels were within normal limits. Imaging studies, including Doppler ultrasonography of the bilateral adrenal glands and magnetic resonance imaging (MRI) of the pituitary gland, revealed no structural abnormalities. Detailed baseline urinary parameters are summarized in Table 1.

Approximately 7 h following the combined administration of arginine and levodopa, the patient developed macroscopic hematuria accompanied by dysuria. After a 24-hour observation period without symptom resolution, he re-presented to our hospital. Subsequent Urinalysis/urine sediment examination indicated: occult blood (+), protein (+), white blood cells (+), ketones (+), red blood cells 10,820 µL↑. A concurrent urine culture was negative for bacterial infection. Notably, the onset of these laboratory abnormalities was closely temporally associated with the medication administration.

Consequently, alternative etiologies for the hematuria—including underlying renal or urological diseases, urinary tract infections, exercise-induced hematuria, and hematuria attributable to other medications—were systematically ruled out.

An adverse drug reaction assessment using the Naranjo algorithm yielded a score of 7, indicating a “probable” causal relationship between the pharmacological intervention and the adverse event (Supplementary Material S1). Therefore, there is a high likelihood that the hematuria was induced by the combined use of arginine and levodopa. Currently, there are no documented drug interactions between arginine and levodopa in the literature. Notably, all previously reported GH stimulation regimens associated with hematuria involve arginine, and prior literature strongly implicates arginine as the causative agent (detailed in Section 3.2, “Previous Literature Reports”). Thus, in the present case, we are inclined to conclude that arginine is the most probable culprit for the observed hematuria.

### 3.2 Previous literature reports

A systematic literature search was conducted across five major databases: PubMed, Embase, China National Knowledge Infrastructure (CNKI), Wanfang Data, and the VIP Database for Chinese Technical Periodicals (VIP). The search encompassed all articles published from the inception of each database up to November 2025.

Eligible study designs included all types of clinical studies, such as randomized controlled trials, non-randomized controlled trials, cohort studies, single-arm studies, and case reports. Duplicate publications, review articles, and studies with incomplete data were excluded. The target population comprised patients undergoing GH stimulation testing with arginine or levodopa. The primary outcome of interest was the occurrence of hematuria (including both microscopic and macroscopic hematuria) as an adverse event following medication administration. No restrictions were imposed regarding patient sex, ethnicity, or geographic region.

**Figure 1:**
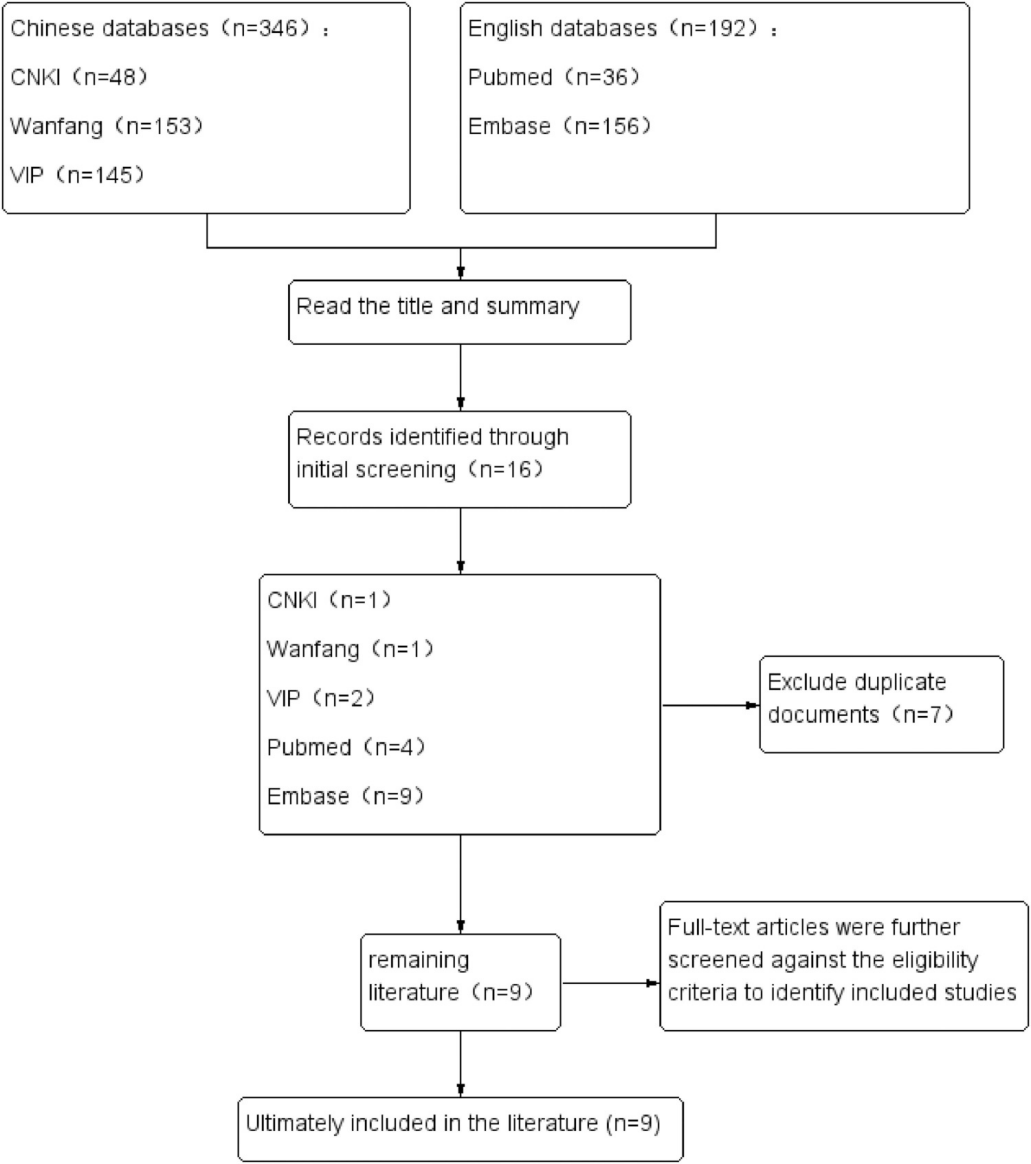
Literature screening process.

The search was limited to literature published in English and Chinese, utilizing combinations of keywords, including “arginine,” “levodopa,” and “hematuria.” The detailed literature selection process is illustrated in Figure 1, and the comprehensive search strategy is provided in Supplementary Material S2.

Ultimately, our search identified 9 publications reporting hematuria associated with arginine-based GH stimulation testing. Of these, 2 publications from China accounted for 4 cases, while 7 international publications (outside China) reported 11 cases [This count explicitly excludes 13 cases from the FDA Adverse Event Reporting System (FAERS) that were merely cited in one of the included studies]. Consequently, a total of 15 fully documented cases of hematuria induced by arginine GH stimulation testing were retrieved.

Notably, only a single literature report described hematuria following a levodopa-based GH stimulation test (involving 1 patient), and this instance also occurred during a combined protocol utilizing arginine. Detailed characteristics of these cases are summarized in Table 2.

**Table 2:** Summary of case reports on hematuria associated with the arginine GH stimulation testing.

Year	Author	Case report: patient characteristics					
		Sex	Age	Drugs (dosage/route)	Onset of hematuria	(1) Characteristics of hematuria. (2) Relevant diagnostic	(1) Management. (2) Outcome
2025	Mostafa et al. (5)	Male	10 years old	Clonidine 90 μg po + Arginine 9 g iv infusion	2 days post-administration	(1) Hematuria accompanied by severe abdominal pain, decreased urinary frequency, presence of blood clots, and burning sensation during urination. (2) Following the onset of hematuria, renal and bladder ultrasonography revealed a bladder distended with blood clots.	(1) Not specified. (2) By day 8, follow-up imaging showed resolution of bladder blood clots and hematuria. A re-examination at 6 weeks was unremarkable.
2022	Xie et al. (6)	Male	7 years old	Levodopa 10 mg/kg po + Arginine 0.5 g/kg iv infusion	2 days post-administration	(1) Uniform gross hematuria throughout voiding, cola-colored or bright red, more pronounced in the morning and alleviated in the evening. Absence of lower urinary tract symptoms (LUTS) such as frequency, urgency, or dysuria. Urinalysis: pH 5, WBC (+++), RBC (+++), Occult blood (+++), Urobilinogen (+++), Bilirubin (++). (2) Post-onset urine culture was negative. Renal and bladder color Doppler ultrasound was unremarkable. Abdominal CT showed suboptimal visualization of the bladder wall with slight thickening.	(1) Urine alkalization and fluid hydration. (2) Gross hematuria gradually resolved within 3 days. Follow-up bladder ultrasound showed complete resolution of flocculent echogenicities, and urinalysis improved. No recurrence of hematuria or complications were noted during a 6-month outpatient follow-up.
2022	Xu et al. (7)	Male	4 years and 7 months old	Clonidine 4 μg/kg po + Arginine 0.5 g/kg iv infusion	2 days post-administration	(1) Uniform gross hematuria throughout voiding, initially pale bloody, without blood clots or LUTS. (2) No significant past medical history; baseline laboratory and imaging studies were unremarkable.	(1) Urine alkalization with sodium bicarbonate. (2) By day 6, the urine cleared with significant clinical improvement.
		Male	5 years and 5 months old		2 days post-administration	(1) Gross hematuria with occasional stringy blood clots, without LUTS. (2) No significant past medical history; baseline tests were normal. Post-onset ultrasonography of the kidneys, bladder, and ureters was unremarkable.	(1) Urine alkalization with sodium bicarbonate; hemostatic therapy with etamsylate and aminomethylbenzoic acid for 3 days. (2) Urine cleared by day 5, and urinary RBC normalized by day 6. Unremarkable at 1-week follow-up.
		Female	4 years and 6 months old		2 days post-administration	(1) Gross hematuria, initially pink and deepening to a port-wine color, without LUTS. (2) No significant past medical history; baseline tests were normal. Post-onset ultrasonography of the kidneys, bladder, and ureters was unremarkable.	(1) Not specified. (2) Gross hematuria cleared by day 5, and microscopic hematuria resolved by day 6. No recurrence during outpatient follow-up.
2021	Elshahawy et al. (8)	Male	9.6 years old	Clonidine po + Arginine iv infusion	2 days post-administration	(1) Gross hematuria. Urinalysis: Protein (3+), Occult blood (3+). (2) Post-onset urine culture was negative; renal ultrasonography was unremarkable.	(1) Conservative management (no specific intervention). (2) Resolved spontaneously within 4 days. Microscopic urinalysis was unremarkable at 1-week follow-up.
2020	Glibbery et al. (9)	Male	5 years and 5 months old	Clonidine 100 μg/m^2^ po + Arginine 0.5 g/kg iv infusion	within 24 h	(1) Severe bilateral calf muscle pain (myalgia) with inability to walk, accompanied by painless gross hematuria. Urinalysis: Occult blood (3+), Protein (1+), Nitrite (−).	(1) Not specified. (2) Myalgia improved by day 2 and fully resolved within 1 week. Gross hematuria also resolved within 1 week. Urinalysis was normal at 2 weeks. No recurrence of myalgia or hematuria during an 11-month follow-up.
2018	Thirunagari et al. (10)	Male	9 years old	Clonidine 4 μg/kg po + Arginine 0.5 g/kg iv infusion	2 days post-administration	(1) Gross hematuria. (2) Post-onset urine culture was negative; renal ultrasonography was unremarkable.	(1) Not specified. (2) Gross hematuria resolved 72 h post-onset.
		Male	9.5 years old		2 days post-administration	(1) Gross hematuria. (2) Post-onset evaluation ruled out infection; abdominal CT scan was unremarkable.	(1) Not specified. (2) Duration unspecified; urinalysis was normal at 2-week follow-up.
2017	Marrone et al. (11)	Unknown	Unknown	Clonidine po + Arginine iv infusion	1 day post-administration	(1) Gross hematuria. (2) Baseline urinalysis prior to administration was normal.	(1) Not specified. (2) Resolved on day 7.
		Unknown	Unknown		1 day post-administration	(1) Microscopic hematuria. (2) Baseline urinalysis prior to administration was normal.	(1) Not specified. (2) Resolved on day 7.
2012	Marinkovic et al. (12)	Male	10.9 years old	Clonidine 5 μg/kg po + Arginine 0.5 g/kg iv infusion	within 48 h	(1) Painless gross hematuria; urinalysis revealed numerous RBCs. (2) Bladder and renal ultrasonography were normal; urine culture showed no bacterial growth.	(1) Not specified. (2) Gross hematuria resolved spontaneously within 6 days.
		Male	11.4岁		2 days post-administration	(1) Transient, painless gross hematuria. (2) Baseline urinalysis was normal; no history of medication use in the preceding 2 months.	(1) Not specified. (2) Hematuria resolved within 2 days of onset.
		Male	6.3 years old		3 days post-administration	(1) Painless gross hematuria; urinalysis showed numerous RBCs. (2) Post-onset urine culture was negative; renal ultrasonography was unremarkable.	(1) Not specified. (2) Urinalysis was normal at 2-month follow-up.
2011	Armada et al. (13)	Male	8.5 years old	Arginine iv infusion	30 h	(1) Gross hematuria without other LUTS (e.g., dysuria or oliguria); urinalysis revealed numerous RBCs. (2) Baseline physical examination and evaluation were normal; post-onset urine culture was negative.	(1) Intravenous fluid hydration. (2) Improvement noted after 4 days; hematuria persisted at 1-month follow-up but showed progressive resolution.
Other reports	A 2020 literature review indicated that the U.S. Food and Drug Administration (FDA) Adverse Event Reporting System (FAERS) recorded 13 cases of hematuria following arginine hydrochloride infusion, although it remains unclear whether these overlap with the aforementioned published cases (12).						

In a small-sample observational study, 34 patients underwent growth hormone (GH) stimulation testing with arginine combined with clonidine, and hematuria developed in three patients following administration of the medications (10). Although the limited sample size may prevent this estimate from accurately reflecting the true incidence, the finding nonetheless suggests that hematuria may represent an underrecognized adverse reaction associated with this testing protocol.

Among the currently reported cases of hematuria, excluding those identified from the FAERS database for which detailed clinical information was unavailable, 12 of the 15 additional cases occurred in male patients, one occurred in a female patient, and the gender of two patients was not specified. The reporting frequency of hematuria appears to be higher in males than in females, suggesting a possible sex-related susceptibility. However, given the limited number of reported cases and the potential for publication bias, the current evidence remains insufficient to draw definitive conclusions regarding sex-specific risk.

The mean age of the 13 pediatric patients was 7.9 years (median, 8.5 years). No clear trend was observed between increasing age and the risk of hematuria, indicating that the occurrence of hematuria does not appear to be strongly correlated with age. Nevertheless, these findings should be interpreted with caution due to the small sample size and the possibility of reporting bias.

Regarding the time of onset, hematuria developed within 1–3 days after arginine GH stimulation testing in 15 cases, with the earliest onset occurring within several hours after the test. In one case, hematuria occurred more than 3 days later. In terms of duration, hematuria resolved within 2 days in one patient and persisted for more than 1 month in another. In the remaining cases, symptoms generally resolved within 4–8 days. Most patients did not exhibit persistent gross hematuria. Overall, recovery typically occurred within approximately 1 week.

With regard to clinical presentation, only one case manifested as gross hematuria accompanied by pain, one case presented with gross hematuria associated with muscle pain, and one case was reported as microscopic hematuria. The remaining cases were characterized as painless gross hematuria. The single case of microscopic hematuria was identified during a clinical study. These findings suggest that hematuria associated with arginine GH stimulation testing is predominantly painless, which may contribute to underrecognition and underreporting of its true incidence, as cases may remain undetected unless routine urinalysis is performed or overt symptoms develop.

Regarding management, some patients received supportive interventions such as urine alkalization and fluid supplementation (6, 7, 13), whereas others were managed conservatively with observation alone (8–10, 12). In all reported cases, hematuria ultimately resolved without long-term sequelae, supporting the conclusion that this condition is generally a self-limiting adverse reaction.

Previous studies have suggested that hematuria occurring after GH stimulation testing is most likely attributable to arginine administration (10). In our review of 15 previously reported cases of hematuria associated with arginine GH stimulation testing, only one case involved the combined use of arginine and levodopa, whereas all other cases involved arginine administered either alone or in combination with clonidine. Based on this distribution, arginine is considered the most likely causative agent in the present case.

Arginine is a conditionally essential amino acid that plays an important role in immune regulation and nitric oxide synthesis in the human body. Common adverse reactions associated with arginine administration include gastrointestinal discomfort and allergic reactions (14, 15). Drug-induced hematuria may result from several pathological mechanisms, including drug-induced nephritis, hemorrhagic cystitis, and eosinophilic cystitis (7).

A previous case report described hematuria following GH stimulation testing with arginine in combination with clonidine. Microscopic examination of the urine revealed erythrocytes of varying sizes, predominantly crenated erythrocytes. Together with findings from urinalysis, renal function testing, and urinary tract ultrasonography, these features were considered consistent with transient benign glomerular hematuria.

Several mechanisms have been proposed to explain arginine-associated hematuria. First, arginine may promote excessive nitric oxide (NO) production *in vivo*, resulting in renal glomerular vasodilation and a transient increase in the permeability of the glomerular basement membrane. Second, a proportion of arginine and its metabolites is excreted through the kidneys and may exert potential nephrotoxic effects, thereby altering the permeability of the glomerular filtration barrier. In addition, children have relatively immature renal function, which may reduce drug clearance and increase the likelihood of drug accumulation. Compared with adults, children also have a thinner glomerular basement membrane and higher glomerular permeability, which may further predispose them to hematuria. Third, the rapid onset and spontaneous resolution of hematuria observed in most cases suggest that immune-mediated mechanisms triggered by drug exposure may also contribute to its development (7). Furthermore, some studies have proposed that arginine infusion may induce intracellular pH alterations, which could potentially result in renal injury (4).

Despite these hypotheses, the precise mechanism underlying arginine-induced hematuria remains unclear. It is likely related to transient alterations in glomerular filtration membrane permeability induced by arginine or its metabolites, or to drug-related immune responses.

## 4 Conclusion

In previously reported cases of hematuria associated with arginine GH stimulation testing, most patients were male, and no clear linear relationship with age was observed. The onset of hematuria generally occurred within 1–3 days after drug administration, and most patients recovered within approximately 1 week. Clinically, the majority of patients presented with changes in urine color alone, while a small proportion experienced associated pain. Management primarily consisted of supportive measures, including urine alkalization and adequate fluid supplementation. Overall, the available evidence suggests that this condition represents a self-limiting adverse drug reaction.

Arginine-induced hematuria is considered a rare adverse reaction. Therefore, prior to initiating GH stimulation testing, clinicians should inform patients and their families about the potential risk of hematuria. In addition, microscopic hematuria may occur after testing and may not be detectable by visual inspection alone. Accordingly, it is recommended that medical institutions with the necessary resources perform post-test urinalysis to monitor for potential microscopic hematuria. Patients should also be advised to observe any changes in urine color and seek medical attention if abnormalities occur.

## Data Availability

The original contributions presented in the study are included in the article/Supplementary Material, further inquiries can be directed to the corresponding author.
