# Supplementary material for: Hematuria following arginine growth hormone stimulation testing in a child: a case report and literature review
Source: Front Pediatr. 2026 Apr 9;14:1763581. doi: 10.3389/fped.2026.1763581 (PMC13102825; doi:10.3389/fped.2026.1763581)
Supplement: Supplementary file 2 [file Supplementaryfile1.pdf]

Attachment 1

The English databases searched in this study included PubMed and Embase. Since the Medline database is included in the PubMed database, it was not searched separately. The following are the search terms for the two databases:

(a)Pubmed:

**Searches**	**Results**
("haematuria"[All Fields] OR "hematuria"[MeSH Terms] OR "hematuria"[All Fields] OR "haematurias"[All Fields] OR "hematurias"[All Fields]) AND ("arginin"[All Fields] OR "arginine"[Supplementary Concept] OR "arginine"[All Fields] OR "arginine"[MeSH Terms] OR "arginine s"[All Fields] OR "arginines"[All Fields] OR "argininic"[All Fields] OR ("levodopa"[Supplementary Concept] OR "levodopa"[All Fields] OR "levodopa"[MeSH Terms] OR "levodopa s"[All Fields]))	36

(b)Embase:

**#**	**Searches**	**Results**
1	'hematuria'	81017
2	'arginine' OR 'levodopa'	257015
3	#1 AND #2	156
