# Supplementary material for: Hematuria following arginine growth hormone stimulation testing in a child: a case report and literature review
Source: Front Pediatr. 2026 Apr 9;14:1763581. doi: 10.3389/fped.2026.1763581 (PMC13102825; doi:10.3389/fped.2026.1763581)
Supplement: Supplementary file 3 [file Supplementaryfile2.pdf]

Naranjo Algorithm - ADR Probability Scale

Naranjo Adverse Drug Reaction Probability Scale Worksheet (PDF – 100 KB)

The Naranjo Algorithm, or Adverse Drug Reaction Probability Scale, is a method by which to assess whether there is a causal relationship between an identified untoward clinical event and a drug using a simple questionnaire to assign probability scores.

Adverse Drug Reaction Probability Scale

Question	Yes	No	Do Not Know	Score
1. Are there previous conclusive reports on this reaction?	+1	0	0	1
2. Did the adverse event appear after the suspected drug was administered?	+2	-1	0	2
3. Did the adverse event improve when the drug was discontinued or a specific antagonist was administered?	+1	0	0	1
4. Did the adverse event reappear when the drug was readministered?	+2	-1	0	0
5. Are there alternative causes that could on their own have caused the reaction?	-1	+2	0	2
6. Did the reaction reappear when a placebo was given?	-1	+1	0	0
7. Was the drug detected in blood or other fluids in concentrations known to be toxic?	+1	0	0	0
8. Was the reaction more severe when the dose was increased or less severe when the dose was decreased?	+1	0	0	0
9. Did the patient have a similar reaction to the same or similar drugs in any previous exposure?	+1	0	0	0
10. Was the adverse event confirmed by any objective evidence?	+1	0	0	1
	**Total Score:**			7

Naranjo Algorithm - ADR Probability Scale

Score	Interpretation of Scores
**Total Score ≥9**	**Definite**. The reaction (1) followed a reasonable temporal sequence after a drug or in which a toxic drug level had been established in body fluids or tissues, (2) followed a recognized response to the suspected drug, and (3) was confirmed by improvement on withdrawing the drug and reappeared on reexposure.
**Total Score 5 to 8**	**Probable**. The reaction (1) followed a reasonable temporal sequence after a drug, (2) followed a recognized response to the suspected drug, (3) was confirmed by withdrawal but not by exposure to the drug, and (4) could not be reasonably explained by the known characteristics of the patient's clinical state.
**Total Score 1 to 4**	**Possible**. The reaction (1) followed a temporal sequence after a drug, (2) possibly followed a recognized pattern to the suspected drug, and (3) could be explained by characteristics of the patient's disease.
**Total Score ≤0**	**Doubtful**. The reaction was likely related to factors other than a drug.

Instructions for Using the ADR Probability Scale

The response "Do not know" should be used sparingly and only when the quality of the data does not permit a "Yes" or "No" answer. "Do not know" can be applicable if the information is not available and also if the question is inapplicable to the case. When more than one drug is involved or suspected, the ADR Probability Scale is usually applied separately to each of the possible etiologic agents, and the drug with the highest score should be considered the causative agent. In addition, the potential of interaction should be evaluated.

Question 1. Are there previous conclusive reports on this reaction? The answer "Yes" (+1) applies if there have been two or more published reports in which the adverse reaction has been described in detail or if the adverse reaction is listed in a reliable source, such as a medical textbook, review article on the medication or on adverse drug reactions, or the product package insert. The response "No" applies when the adverse event has not been described previously or if only one report has been published, or if published reports were considered inconclusive or unconvincing. The answer "Do not know" is applicable only when there is no information, because the agent has not been available for an adequate period of time or has not been

previously evaluated for this adverse reaction. The scores given for “No” and “Do not know” are the same (0), so it is not critical to decide between these two answers.

Question 2. Did the adverse event appear after the suspected drug was administered? This question evaluates the temporal relationship between the reaction and administration of the medication. The answer “Yes” (+2) applies if there is definitive evidence that the adverse event occurred after the medication was started. “No” (-1) applies when the adverse event developed before the first dose of the drug. “Do not know” (0) applies if the information is not available or is unclear.

Question 3. Did the adverse event improve when the drug was discontinued or a specific antagonist was administered? This question evaluates the response to dechallenge or stopping the medication. The answer “Yes” (+1) applies if the adverse event diminishes or disappears at any time after stopping the medication, or if the reaction disappears upon administration of a specific pharmacologic antagonist (for example, an anticholinergic given for a cholinergic reaction to physostigmine). The answer “No” (0) applies if the adverse event does not improve or improves in response to a nonspecific therapy or an antidote to another medication or treatment of the underlying disease. The answer “Do not know” (0) applies if the medication was not stopped or the subsequent course was unknown, inconclusive or unclear.

Question 4. Did the adverse event reappear when the drug was readministered? This question evaluates the response to rechallenge or reexposure. An answer of “Yes” (+2) indicates that the medication was stopped, the adverse event resolved or improved, and there was an unequivocal reappearance or worsening of the reaction when the medicine was restarted in a similar dose and by the same route. The Naranjo scale also allows for a “Yes” if the causal association is well known and rechallenge cannot be done for clinical or ethical reasons. An answer of “No” (-1) only applies if rechallenge was done, but the adverse event did not reappear or worsen. The answer “Do not know” (0) applies if rechallenge was not done or information on rechallenge is not available or the reaction was ambiguous.

Question 5. Are there alternative causes that could on their own have caused the reaction? This question assesses alternative explanations for the adverse event. Because adverse events are often nonspecific and can be manifestations of the disease being treated or an unrelated, concurrent disease or condition, other diagnoses need to be considered and excluded. The answer “No” (+2) applies if alternative causes have been excluded, based upon a systematic and complete evaluation, thus implicating the drug more strongly. A risk or susceptibility factor is not an alternative cause. The answer “Yes” (-1) applies when there is an alternative cause or explanation. “Do not know” (0) applies if the investigation of other causes is incomplete, inconclusive or was not done.

Question 6. Did the reaction reappear when a placebo was given? This question applies to clinical research studies in which a placebo was administered. The answer “Yes” (-1) applies if the medication was stopped and the adverse reaction resolved or improved conclusively, and there was an unequivocal reappearance of the adverse event after administration of placebo (single or double blind). The answer “No” (+1) applies if the reaction did not reappear or worsen after administration of placebo. “Do not know” (0) applies if placebo challenge was not done or the results were inconclusive.

Question 7. Was the drug detected in blood or other fluids in concentrations known to be toxic? This question applies specifically to dose dependent adverse reactions when blood, urine, tissue or other specimen concentrations of the medicine are available. The answer “Yes” (+1) applies if the concentration is in the accepted toxic or supratherapeutic range. “No” (0) applies if the concentration is below the toxic range. The answer “Do not know” (0) applies if drug levels are not available or are inconclusive.

Question 8. Was the reaction more severe when the dose was increased or less severe when the dose was decreased? This question evaluates the dose response relationship of medication and the adverse reaction. “Yes” (+1) applies if the adverse event was more severe or worsened when the dose of the medication was increased, or was less severe and improved when the dose was decreased. “No” (0) applies if there was no appreciable change in the severity of the adverse event with dose modification. “Do not know” (0) applies if the dose or regimen was not altered or the information was not available or inconclusive.

Question 9. Did the patient have a similar reaction to the same or similar drugs in any previous exposure? This question is directed at past medical history of adverse reactions to the same or a structurally related drug. “Yes” (+1) applies when there is documentation of a previous similar reaction to the specific drug or a related medication. “No” (0) applies when the patient does not have a previous exposure to the same medicine or when the patient did not develop the adverse reaction in a previous exposure to the same or related drugs. “Do not know” (0) applies when there is no information on previous reactions or the information is inconclusive.

Question 10. Was the adverse event confirmed by any objective evidence? The final question assesses the quality of the data on which the adverse event is assessed. “Yes” (+1) indicates that there is laboratory test documentation of the adverse event or that the event was directly observed by a qualified person (for example, a skin rash described in nursing or physician notes). The answer “No” (0) applies when neither laboratory tests nor direct clinical documentation can verify the reaction. “Do not know” (0) applies if there is no specific information available (no laboratory testing and no clinical description) or the information is inconclusive. The scores given for “No” and “Do not know” are the same (0), so it is not critical to decide between these two answers.

Selected References

- Naranjo CA, Busto U, Sellers EM, Sandor P, Ruiz I, Roberts EA, Janecek E, et al. A method for estimating the probability of adverse drug reactions. Clin Pharacol Ther. 1981;30:239–45. [PubMed]

  (Initial description of Naranjo system for assessing adverse drug reactions, giving -1 to +2 points for 10 items: prevous conclusive reports, time of onset, improvement on stopping, reappearance with reexposure, alternative causes, reappearance with placebo, drug levels, dose response, previous exposure and history, and confirmation by objective evidence with total scores that range from -4 to +13; kappa=0.64 to 0.71 in assessing 63 cases by 3 authors).
